# Comparison of the egg recovery rates and limit of detection for soil-transmitted helminths using the Kato-Katz thick smear, faecal flotation and quantitative real-time PCR in human stool

**DOI:** 10.1371/journal.pntd.0009395

**Published:** 2021-05-26

**Authors:** Patsy A. Zendejas-Heredia, Vito Colella, Sze Fui Hii, Rebecca J. Traub

**Affiliations:** Faculty of Veterinary and Agricultural Sciences, University of Melbourne, Parkville, Victoria, Australia; NIH-National Institute for Research in Tuberculosis-ICER, INDIA

## Abstract

**Background:**

Monitoring the success of soil-transmitted helminth (STH) control programs relies on accurate diagnosis and quantitative assessment of infection prevalence and intensity. As preventative chemotherapeutic program coverage for STH expands, the necessity of gaining insights into the relative or comparative sensitivities, in terms of limits of detection (LOD) and egg-recovery-rates (ERR) for microscopy and quantitative polymerase chain reaction qPCR-based diagnostic techniques becomes imperative to inform suitability for their intended use for large scale STH monitoring and treatment efficacy studies.

**Methodology/Principal findings:**

The diagnostic performance in terms of ERR and LOD of the Kato-Katz (KK) thick smear technique, sodium nitrate (NaNO_3_) faecal floatation (FF) and qPCR for the accurate detection and enumeration of STH eggs were calculated and expressed in eggs per gram (EPG), by experimentally seeding parasite-free human faeces with *Ascaris* spp., *Trichuris* spp. and *Necator americanus* eggs representing low, medium and high intensity infections. The efficiency of NaNO_3_ flotation was also calculated over a range of specific gravities (SpGr) for the optimum recovery of STH eggs. FF of SpGr 1.30 recovered 62.7%, 11% and 8.7% more *Trichuris* spp., *Necator americanus* and *Ascaris* spp. eggs respectively, than the recommended SpGr of 1.20. All diagnostic methods demonstrated strong direct correlation to the intensity of seeded EPG. KK and FF (SpGr 1.30) resulted in significant lower ERRs compared to qPCR (*p* <0.05). qPCR demonstrated significantly (p <0.05) greater sensitivity with an ability to detect as little as 5 EPG for all three STH, compared to 50 EPG by KK and FF (SpGr 1.30).

**Conclusions/Significance:**

This study compares the diagnostic parameters in terms of LOD and ERRs of STHs for the KK, FF and qPCR. These results indicate that the diagnostic performance of qPCR assays should be considered by control programs in the phase that aims to seek confirmation of transmission break and cessation of preventive chemotherapy in low-transmission settings, in line with the control targets of the WHO neglected tropical diseases 2030 Roadmap.

## Author summary

STH infections predominately affect resource-poor communities and negatively impact on child and maternal health. Diagnostics play a critical role in guiding and informing existing STH control programs and the implementation and evaluation of intervention strategies. The KK technique is most commonly used as per WHO guidelines and is the basis for determining quantitative thresholds for low, moderate and heavy infections. More recently, FF and laboratory-based quantitative PCR techniques have provided alternative options of diagnosing STH infections. A number of studies correlating the relative sensitivity, diagnostic agreement, and egg enumeration of these techniques by comparing field-generated diagnostic data have attempted to correlate quantitative outputs to those defined by the WHO, with little resolution. Moreover, as large-scale deworming programs levels scale up, the necessity to apply techniques that can precisely detect light infections is of outmost importance to establish preventive chemotherapy end-points using more accurate means. In this study we compared the LOD and ERR of seeded non-infected human faecal samples with *Ascaris*, *Trichuris* and *Necator* spp. eggs using KK, FF and qPCR. When compared to copro-microscopy methods, qPCR showed the highest sensitivity for the detection of light-intensity infections and was more accurate in enumerating the original number of eggs per gram of seeded faeces. In addition to this, the results indicated that the diagnostic performance of both KK and FF (using solution at a SpGr of 1.30) were equally efficient and should be considered with their diagnostic limitations in mind, when aiming to monitor STH infections in low-transmission settings.

## Introduction

Over 1.5 billion people are infected with at least one soil-transmitted helminth (STHs; *Ascaris lumbricoides*, *Necator americanus*, *Ancylostoma* spp. and *Trichuris trichiuria*) worldwide. Children, pregnant women and women of child-bearing age from the poorest and most marginalized communities are severely impacted in terms of morbidity [1]. The World Health Organization (WHO) endorsed a resolution to eliminate morbidity caused by the STHs by 2020 through periodical deworming of all at-risk populations in endemic areas [2] and has now set new global targets to build on this target by 2030 [3]. The current global control strategy aims to control morbidity of STHs through large-scale deworming programs using preventive chemotherapy (PC; albendazole or mebendazole) for all at-risk populations (pre-school and school children, women of reproductive age). To date, 50.25% of the world population still requires PC. As STH morbidity and transmission are directly related to the infection prevalence and intensity [4], accurate diagnosis and quantitation of infections is paramount for assessing impact and informing control programs. More precisely, the sensitivity of diagnostic techniques becomes critical in later stages of PC programs, when infection prevalence and intensity decrease, to identify areas of low-transmission and make informed decision on PC interruption [5].

The diagnosis and measure of morbidity attributed to STHs currently relies on the detection and quantitation of eggs in faeces through the assessment of faecal egg counts (FEC; expressed in eggs per gram of stool or EPG). The Kato-Katz (KK) thick smear is the most widely utilized and accepted technique recommended by the WHO and the thresholds for low, moderate and heavy infections have traditionally been based on this technique [6,7]. The technique is simple, inexpensive, reproducible and commonly used in field-based epidemiological surveys. However, a major critique of the KK is its reduced sensitivity in detecting low intensity infections, its predisposition to false negative results and the need for several slides per sample to obtain accurate FEC [5,8–10]. Alternatively, coproscopy-based flotation methods such as the simple or centrifugal faecal float (FF), FLOTAC, mini-FLOTAC, FECPAK, McMaster and most recently DNA based diagnostic methods such as quantitative PCR (qPCR) have demonstrated advantages over KK[10–12]. FF has been demonstrated superior to KK performed in duplicate and quadruplicate for detecting light intensity infections [10,11]. On the other hand, the egg intensity levels as determined by FF using a floatation solution with a specific gravity (SpGr) of 1.20, have tended to produce on average, lower values compared to those of KK [10,13]. FF like KK is inexpensive and provides clean preparations that allow clear observation of ova.

A number of studies have reported the superiority of qPCR compared to microscopy-based methods in terms of sensitivity, its ability to detect higher levels of mixed STH species infections and ability to identify hookworm eggs at species level [12,14,15]. This is important given the emerging distribution of the zoonotic hookworm *Ancylostoma ceylanicum* [14,16].

Despite the availability of literature assessing the field-based comparative sensitivities of the aforementioned STH diagnostic techniques [11,12,17–20] to our knowledge, no data exists on the assessment of the true ERR and/or limit of detection (LOD) of three STHs by KK, FF and qPCR in human stool. In this study we report a comparison of the ERR and LOD of seeded *Ascaris*, *Trichuris and Necator* eggs in parasite-free human faeces using KK, sodium nitrate (NaNO_3_) FF (SpGr 1.20, 1.25, 1.30 and 1.35), and multiplex qPCR that quantifies genera- and species-specific infection intensities using a pre-determined cycle-threshold to EPG formula.

## Methods

### Egg purification

Given difficulties in obtaining fresh eggs from infected humans and the similarities in egg morphology between human (*T*. *trichiuria* and *A*. *lumbricoides*) and swine STH, we used gravid *A*. *suum* female worms and *T*. *suis* positive faeces sourced from a local pig abattoir in Victoria, Australia. Unmatured eggs of *Ascaris* were recovered by mechanical dissection of the uterus of an adult female worm followed by repeatedly filtering eggs through a double layer of 10 cm sq surgical gauze with 1x PBS. 10 μL of solution with eggs was quantified in triplicate using light microscopy. *Ascaris* eggs (n = 1000) were checked in triplicate prior seeding to ensure eggs were fertilized and not unfertilized to avoid egg ploidy. *N*. *americanus* eggs were sourced from fresh human stool provided by Prof. Alex Loukas (James Cook University, Qld, AUS). Unmatured eggs of *N*. *americanus* and *Trichuris* were purified from human and pig faeces using gradient centrifugal flotation using Sheather’s solution (1.20 SpGr; 355ml distilled water (dH2O) and 454 g sucrose). Briefly, 3–5 g of faeces were strained through surgical gauze using 20 ml of dH20 and transferred into a 50 ml centrifuge tube. The filtrate was centrifuged for two minutes at 2000 x rpm. The supernatant was discarded leaving behind the faecal pellet. Faeces were homogenized with Sheather’s solution using a wooden stick, filled to the rim of the centrifuge tube and allowed to sit for 15 min. The top layer was carefully aspirated and placed into 15 ml centrifuge tubes followed by a washing step with 1x PBS and centrifugation at 2000 rpm for 5 min. Following removal of the supernatant,10 μl of sediment consisting of purified eggs was quantified (eggs/μl) in triplicate using light microscopy. All purified egg species were identified using previous molecular methods [14,21,22] and stored at 4°C. Eggs were microscopically checked to avoid inclusion of samples in which egg embryonation occurred.

### Stool collection and parasitological procedures

Stool samples were sourced from an anonymous donor living in Victoria, Australia, with no history of travel to a STH-endemic area. Vials (50 ml) with popsicle sticks were provided to the participant and once collected, examined by FF and qPCR to ensure the sample was parasite-free prior to the egg seeding experiment. STH-free stool samples were placed into 15 ml centrifuge tubes for KK and FF (1gr each) and into 1.5 ml Eppendorf tubes (200 mg each) for qPCR. A range of infection intensities, measured by EPG was selected to be seeded into faecal samples in triplicate based on current WHO classifications of light, moderate and heavy STH infection intensities [23]. Between 1–8,000 eggs of *Necator spp*., 1–15,000 eggs of *Trichuris spp*. and 1–50,000 eggs of *Ascaris spp*. were seeded in triplicate into each STH-free stool sample within individual tubes, as detailed in Table 1.

**Table 1:** Serial replicates (n = 3) of soil-transmitted helminth eggs spiked in one gram of parasite-free faeces according to infection intensities selected from WHO guidelines.

*Trichuris spp*.	*Ascaris spp*.	*Necator americanus*
**Low intensity infections**		
1–999*	1–4,999*	1–1999*
5	5	5
10	10	10
50	50	50
100	100	100
500	150	500
	500	1,000
	2,000	1,500
**Moderate-Intensity infections**		
1,000–9,999*	5,000–49,999*	2,000–3,999*
1,000	5,000	2,000
2,000	10,000	3,000
5,000	25,000	
7,500		
	**High-intensity infections**	
≥10,000*	≥50,000*	≥4,000*
10,000	50,000	4,000
15,000		6,000
		8,000

* Classes of infection intensities for soil-transmitted helminths as per WHO guidelines.

### Kato-Katz procedure

For each stool sample (n = 3), KK smears (Sterlitech, USA) were performed in duplicate as per WHO’s recommendations [7]. The slide was examined by light microscopy within 60 min following preparation and the number of *Ascaris*, *Trichuris* and *Necator* eggs enumerated. The absolute egg count for each triplicate were summed and multiplied by 12 to obtain EPG value for each seeded stool sample.

### Specific gravity for optimal egg recovery using sodium nitrate solution

To assess the optimal SpGr for achieving the highest egg recovery yield by standing FF, the ERR for each parasite species was determined by subjecting one gram of STH-egg seeded stool (100 EPG for each *Necator*, *Ascaris* and *Trichuris*) to flotation using NaNO_3_ solution with a range of SpGr (i.e. 1.20, 1.25, 1.30 and 1.35, in triplicate).

Once optimised, FF was carried out previously described [24], with parasite-free samples inoculated with a range of egg concentrations for each parasite species in triplicate as shown in Table 1, using a double cover slip method. Briefly, one gram of faeces was homogenized with NaNO_3_ and strained through a 10 cm sq surgical gauze into a sterile specimen container. The suspension was transferred to a 15 ml centrifuge tube and centrifuged for 5 min at 2500 × rpm. The remaining faecal material remaining was recorded for further EPG calculation. Further NaNO_3_ was added to the rim of the tube, forming a positive meniscus and allowed to stand for 10 min with a coverslip (22 mm × 22 mm). Once removed, a second coverslip was placed and allowing to stand for a further 5 min after which both coverslips were placed on a slide and examined at 100 magnification by light microscopy. The entire slide was examined in a zig-zag fashion and the total number of eggs was recorded.

ERR were calculated on the basis of total numbers of eggs recovered per gram and number of eggs seeded (for detailed protocol see S1 File).

### Generation of formula to convert Ct values to EPG for STH

Genomic DNA was extracted from each triplicate individually (200mg of stool) containing serial dilution of known intensity of seeded eggs of *Ascaris*, *Necator* and *Trichuris* using a Maxwell RSC PureFood GMO and Authentication Kit, Promega (Promega Corporation, US) as per manufacturer’s instructions, with the following modifications; an additional bead-beating step with 400μL CTAB buffer using a FastPrep-24 5G Instrument, (MP Biomedicals) and 0.5 mm Zirconia/Silica beads (Daintree Scientific, AUS). Following bead-beating, cell lysis was proceeded in a Maxwell RSC 48 Instrument, Promega. The final eluted sample (100 μL) was stored at -20°C for further downstream analyses. DNA samples were subjected to two multiplex qPCR (M-qPCR): 1. Hookworm-human four-plex qPCR; 2. *Ascaris*-*Trichuris* four-plex qPCR. Details of the probes and primers are listed in Table 2. Both qPCR assays were performed in triplicate for each individual sample using Taq Man hydrolysis probes (Integrated DNA Technologies, USA) in a Mic qPCR Cycler system (Bio molecular systems, AUS). Assays were performed in 20 μl reactions containing 10 μl of GoTaq Probe qPCR Master mix (Promega Corporation, USA), 1 μL of known quantity of EHV4 DNA as an internal qPCR control, and 2 μL of template DNA. Nuclease free-water was added to reach the final reaction volume. Non-template controls (assay master-mix with no template) were included with each run. The cycling conditions for both assays consisted of the following parameters: denaturation at 95°C for 2 min, followed by 40 cycles of 15 sec at 95°C and annealing at 60°C for 1 min, with no extension phase. Egg counts expressed in EPG were calculated by multiplying the absolute egg count by a factor of 5, as 200 mg of faecal sample was subjected to DNA extraction for the current study. Log10 transformations of original egg count (EPG) for each STH species were plotted against Ct values to predict the ability of the assay to estimate original egg intensities in EPG for each STH species, assuming a 100% run efficiency [14].

**Table 2:** Quantitative multiplex PCR oligonucleotide primers and probes for the detection of soil-transmitted helminths.

Multiplex qPCR	Target species	Oliglonucleotide sequence 5’-‘3	Product size	Gene target	Final conc. in nM	Source
Ascaris-Trichuris-EHV4 qPCR	*Ascaris* spp.		88 bp	ITS1		
	Asc Fwd	GTAATAGCAGTCGGCGGTTTCTT			50	[25]
	Asc Rev	GCCCAACATGCCACCTATTC			50	
	Asc Probe	/5HEX/TT GGC GGA C/ZEN/A ATT GCA TGC GAT /3IABkFQ/			100	
	*Trichuris* spp.		76bp	18S		[26]
	Tri 18 S Fwd	TTGAAACGACTTGCTCATCAACTT			250	
	Tri 18 S Rev	CTGATTCTCCGTTAACCGTTGTC			250	
	Tri 18S Probe	/CY5 -CGATGGTAC/TAO/GCTACGTGCTTACCATGG- 3IAbRQSp/			100	
	Human DNA					
	Mammal F	CGACCTCGATGTTGGATCAG	92bp	16S	50	This study
	Mammal R	GAACTCAGATCACGTAGGACTTT			50	
	Human Probe	/FAM/CCCGATGGT/ZEN/GCAGCCGCTATTAAA /3IABkFQ/			100	
	Equine Herpes Virus		81 bp	gB		[12]
	EHV Fwd	GATGACACTAGCGACTTCGA			40	
	EHV Rev	CAGGGCAGAAACCATAGACA			40	
	EHV probe	/ROX/TTTCGCGTGCCTCCTCCAG/3IAbRQSp/			100	
Hookworm qPCR	*Necator americanus*		101 bp	ITS2		[27]
	Nec Fwd	CTGTTTGTCGAACGGTACTTGC				
	Nec Rev	ATAACAGCGTGCACATGTTGC				
	Nec Probe	/5Cy5/CTG+TA+CTA+CG+CAT+TGTATAC/3IAbRQSp/*				
	*Ancylostoma* spp.					[14]
	Anc F	CGGGAAGGTTGGGAGTATC	104 bp	ITS1	300	
	Anc R	CGAACTTCGCACAGCAATC			300	
	A.cey Probe	/56-FAM/CCGTTC+CTGGGTGGC/3IABkFQ/			100	
	A.duo Probe	/5HEX/TCGTTAC+T+GGGTGACGG/3IABkFQ/			100	
	Equine Herpes Virus		81 bp	gB		[12]
	EHV Fwd	GATGACACTAGCGACTTCGA			40	
	EHV Rev	CAGGGCAGAAACCATAGACA			40	
	EHV probe	/ROX/TTTCGCGTGCCTCCTCCAG/3IAbRQSp/			100	

### Multiplex STH qPCRs

Genomic DNA was extracted as per previously described and the seeded stool samples containing *Ascaris*, *Necator* and *Trichuris* eggs representing light, moderate and heavy infection intensities (Table 1) subjected to the two four-plex qPCR assays in duplicate 1. Hookworm-human four-plex qPCR; 2. *Ascaris*-*Trichuris*- four-plex qPCR assays as previously described. EPG for each sample were estimated using the Ct value to EPG formula generated.

### Data analysis

Analyses were performed using GraphPad Prism version 8.4.2 for Windows (GraphPad Software, La Jolla California USA, http://www.graphpad.com) and Excel 2017 (Microsoft).

### Specific gravity for optimal egg recovery using sodium nitrate solution

The ability of NaNO_3_ to recover *Ascaris*, *Trichuris* and hookworm eggs from human faeces was compared using a range of SpGrs from 1.20–1.35. Absolute FECs were recorded and transformed to EPG using the equation to convert FECs to EPG from FF. Descriptive statistics was conducted to observe the distribution of the data and obtain the geometric mean of each SpGr and their respective 95% confidence intervals. Ordinary One-way ANOVA test was used to compare the overall ERR means of the SpGrs tested. A *p*-value of less than 0.05 was required for significance. Because the overall test was significant, a post-hoc test using Tukey’s multiple comparisons, with a single pooled variance between SpGr means was conducted to assess the point or level of difference for which a significant difference laid.

### Diagnostic performance of qPCR and microscopy techniques

Normality and lognormality tests and frequency distribution of the data was evaluated following Shapiro-Wilk normality test for each parasite, with a significance level of α = 0.05. Agreement in EPG between the seeded experiment and the ERR for each technique was evaluated by the non-parametric Spearman’s rank correlation coefficient (ρ). In addition, the agreement between each technique excluding seeded EPG was further evaluated for pair-wise comparisons. Descriptive statistics were conducted to generate data on the ERR frequency of each of the techniques followed by one-way ANOVA to test for the significance of the ERR variability among means. Tukey’s multiple comparison test was used to further analyse the ERR performance of each technique. A *p* value of 0.05 was required for significance.

## Results

### Specific gravity for optimal egg recovery using sodium nitrate solution

**Fig 1:**
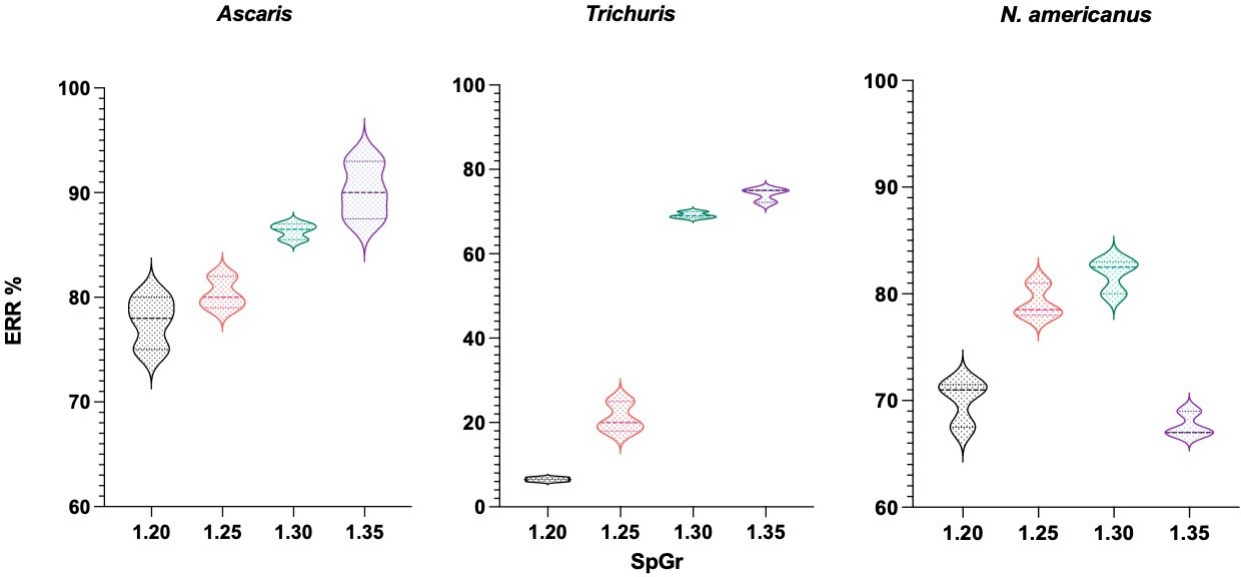
Violin plots for egg recovery rates with sodium nitrate faecal floatation at different specific gravity concentrations. Empirical distributions of data sets obtained from triplicate faecal float counts of 100 seeded *Ascaris*, *Trichuris* and *N*. *americanus* eggs in parasite-free faeces using four specific gravity concentrations of sodium nitrate solution.

A range of SpGrs was tested to compare the absolute number of eggs recovered using NaNO_3_ from 100 seeded *Ascaris*, *Trichuris* and *Necator* eggs in 1 gr of human parasite-free faeces. One-way ANOVA analyses revealed a significant difference among the ERR means for the different SpGrs tested for *Ascaris* (*p* = 0.003, R^2^ 0.90), *Trichuris* (p <0.001, R^2^ 0.99) and *N*. *americanus* (p <0.001, R^2^ 0.95) (Table 3). High F-values were observed for the three parasites (*Ascaris* F = 22.92; *Trichuris* F = 843; *N*. *americanus* F = 50.63) indicating evidence for differences among means. Mean ERRs for each species across different SpGrs and their respective Tukey’s test values are reported in Table 4. The empirical distribution of the data for each parasite is shown in Fig 1. In general, ERR increased as the SpGr increased for the three STHs (Table 4). *Ascaris* ERR’s increased from 77.6% using 1.20 SpGr to 90.3% when using a SpGr of 1.35. However, only a SpGr increase from 1.25 to 1.30 provided a significant higher *Ascaris* ERR (*p* = 0.025) from 80.3% to 86.3%. SpGr of 1.20 and 1.30 resulted in comparable ERRs for *N*. *americanus* of 70.1% and 81.8% (p 0.007), respectively. However, ERR decreased significantly (*p =* 0.003*)* for *Necator* when the SpGr was increased from 1.30 (81.8%) to 1.35 (67.7%). *Trichuris* egg recovery rates increased from 6.5% using a 1.20 SpGr to 69.2% with a 1.30 SpGr solution (p 0.006). Thus, a SpGr of 1.30 was deemed optimum for maximizing the ERR for all STHs.

**Table 3:** ANOVA for egg recovery rates from *Ascaris* spp., *Trichuris* spp. and *N*. *americanus* 100 seeded eggs using 1.20, 1.25, 1.30 and 1.35-SpGr NaNO_3_.

Parasite	Source of variation	Sum of squares	Degrees of freedom	Mean sum of squares	F (DFn, DFd)	*P value*	*R*^*2*^
***Ascaris***							
	Between SpGrs	289.4	3	96.47	F (3, 8) = 22.92	0.003*	0.90
	Within SpGrs	33.67	8	4.208			
	Total	323.1	11				
***Trichuris***							
	Between SpGrs	10397	3	3466	F (3, 8) = 843	<0.001***	0.99
	Within SpGrs	33	8	4.1			
	Total	10430	11				
***N*. *americanus***							
	Between SpGrs	427.2	3	142.4	F (3, 8) = 50.63	<0.001***	0.95
	Within SpGrs	22.50	8	2.813			
	Total	449.7	11				

*P<0.05

**P<0.002

***P<0.001

**Table 4:** Egg recovery rate (ERR) comparison from triplicate absolute faecal egg counts of 100 seeded *Ascaris* spp., *Trichuris* spp. and *N*. *americanus* eggs.

Parasite	1.20 SpGr		1.25 SpGr		1.30 SpGr		1. 35 SpGr
	ERR mean % [95% CI]	*p-value* 1.20–1.25	ERR mean % [95% CI]	*p-value* 1.25–1.30	ERR mean % [95% CI]	*p-value* 1.30–1.35	ERR mean % [95% CI]
***Ascaris***	77.6 [71.4; 83.9]	0.409	80.3 [76.5; 84.1]	0.025*	86.3 [84.4; 88.2]	0.145	90.3 [83.9; 96.7]
***Trichuris***	6.50 [5.26; 7.74]	0.047*	21 [12.0; 29.9]	0.006*	69.2 [67.3; 71.1]	0.088	74.1 [70.0; 78.1]
***N*. *americanus***	70.1 [65.1; 75.1]	0.007*	79.2 [75.4; 83.1]	0.285	81.8 [77.8; 85.8]	0.003*	67.7 [64.8; 70.5]

*P<0.05

**P<0.002, ***P<0.001

*α =* 0.5 for every 0.5 SpGr increase from Tukey’s multiple comparison test from two-way ANOVA

### Relationship between known numbers of STH seeded eggs and Ct values from multiplex STH qPCRs

**Fig 2:**
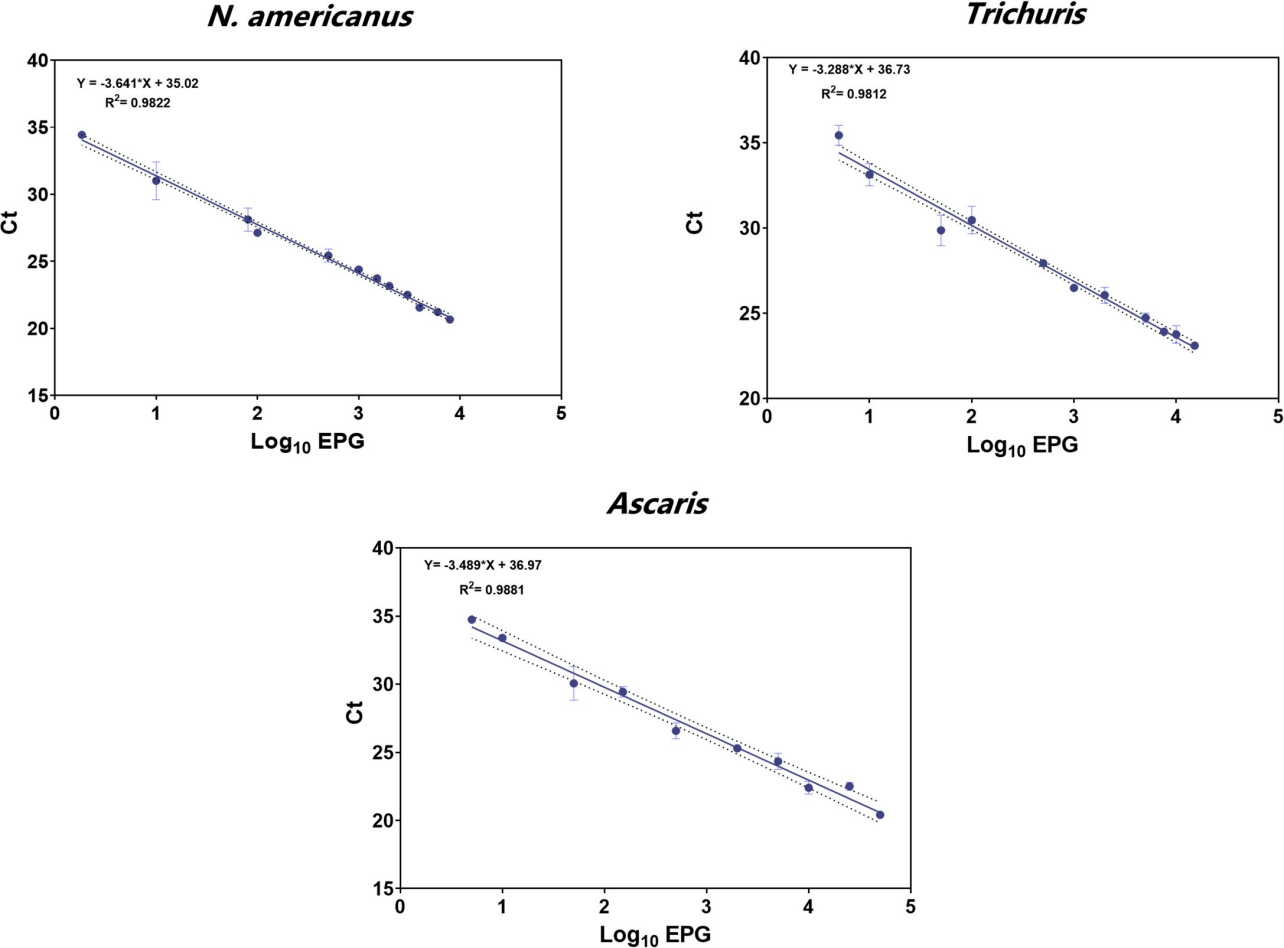
Agreement in eggs per gram (EPG) and cycle threshold (Ct) values by qPCR. The scatterplots illustrate a strong agreement (*Ascaris*: R^2^ = 0.9881; *Trichuris*: R^2^ = 0.9812; *N*. *americanus*: R^2^ = 0.982) between faecal egg counts (FECs) expressed as EPG of stool from seeded known numbers of *Ascaris*, *Trichuris* and *N*. *americanus* eggs and the mean Ct values obtained from qPCR reads from two multiplex qPCR assays. Dotted lines adjacent to the best line of fit represent 95% confidence intervals; graph error bands indicate the standard error of the plotted means (SEM) which measures the variability/dispersion of the triplicate Ct values.

Quantitative results derived from well-defined series of purified known seeded STH eggs (Table 1) plotted against Ct values for *Ascaris*, *Trichuris* and *N*. *americanus* are shown in Fig 2. The interpolation of qPCR Ct-value to log10 EPG for the three-helminth species showed a strong linear relationship between the two variables (*Ascaris* R^2^ = 0.988, *Trichuris* R^2^ = 0.981 and *N*. *americanus* R^2^ = 0.982). The conversion formulas for Ct to EPG were determined as the following:

$$EPG\;Ascaris=10^{(Ct-36.97)/-3.489}$$

$$EPG\;Trichuris=10^{(Ct-36.73)/-3.288}$$

$$EPG\;N.americanus=10^{(Ct-35.02)/-3.641}$$

### Diagnostic performance of qPCR and microscopy techniques

**Fig 3:**
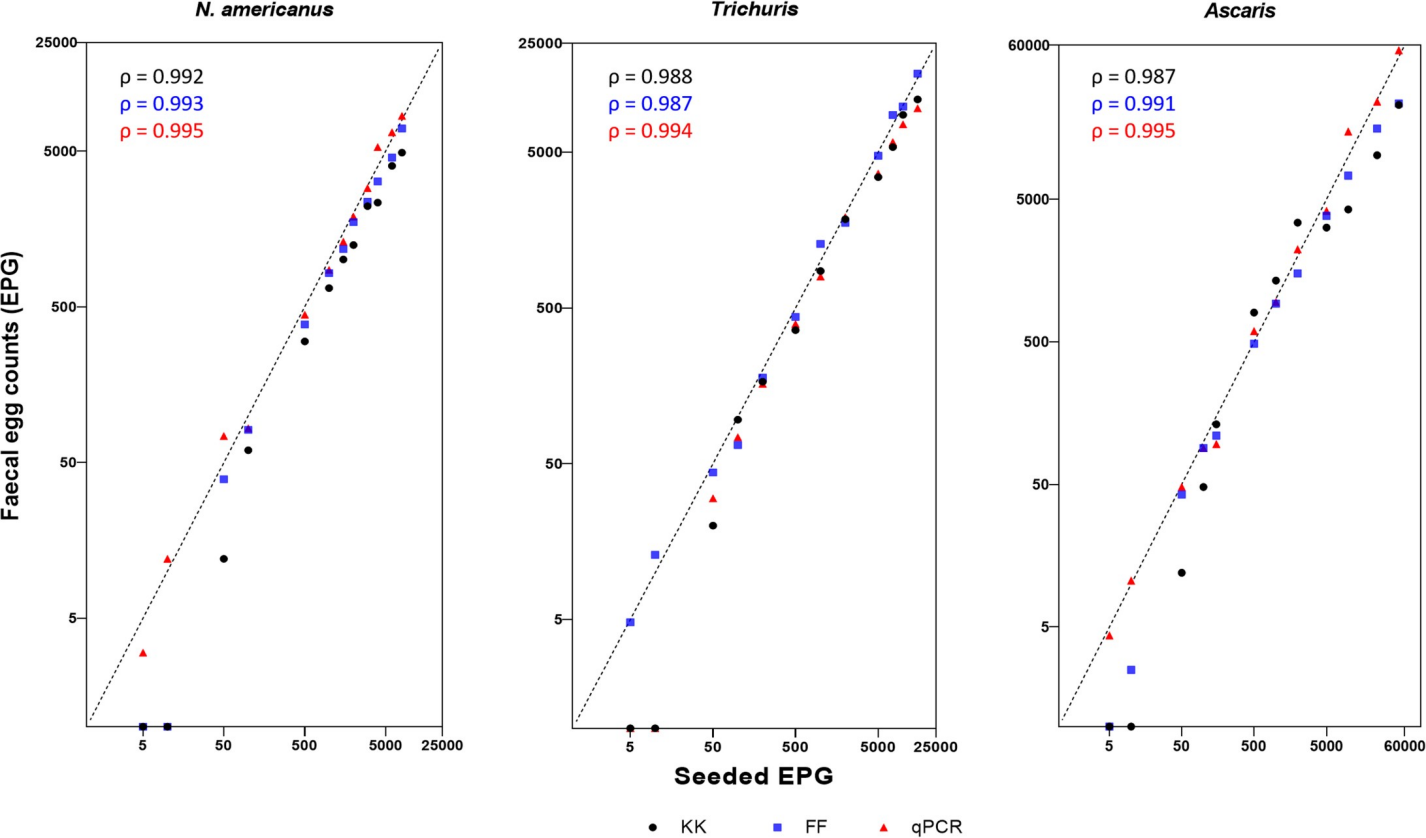
Agreement of faecal egg counts by the two microscopy techniques, qPCR and seeded EPG. The scatter plots illustrate the agreement between faecal egg counts, expressed in eggs per gram (EPG) based on each technique versus known numbers of seeded eggs per gram of stool. Black dots (Kato Katz); blue squares (Faecal floats); red triangles (qPCR). In each panel, the Spearman’s rank correlation coefficient (ρ) is given and a diagonal stripped line represents the line of equivalence.

**Fig 4:**
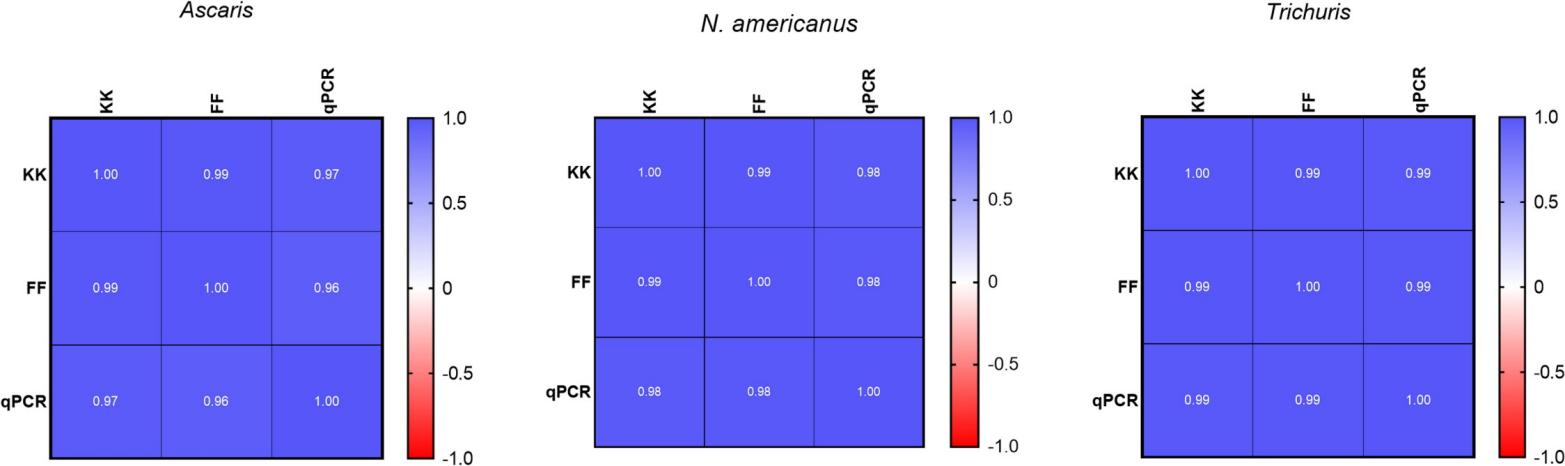
Pairwise correlation matrix heatmaps of each diagnostic technique for each of the STHs. Each square illustrates the correlation between the performance of each technique. Correlation ranges from -1 to +1 (values closer to zero means there is no linear correlation between the variables examined). The plot is symmetrical on the diagonal since the same two techniques are being paired together in that same square.

The data distribution of each parasite failed to follow a Gaussian distribution based on the Shapiro-Wilk test for normality (*p* <0.05). Agreement in recovered EPG from the two microscopy techniques, and qPCR compared to the seeded EPG for each STH using the Spearman’s rank correlation are shown in Fig 3. Overall, a strong positive linear correlation was observed between ERR and seeded EPG for the three diagnostic techniques among all STHs. The highest correlation coefficient was between qPCR and seeded EPG (ρ = 0.994–0.995, *p* <0.001), followed by FF (ρ = 0.987–0.993, *p* < 0.001) and KK (ρ = 0.987–0.992 *p* < 0.001). The highest correlation coefficient for each pair-wise comparison between diagnostic techniques was observed between FF vs KK (R_s_ >0.99) followed by KK vs qPCR (R_s_ = 0.97–0.99) and qPCR vs FF (R_s_ = 0.96–0.99) (Fig 4). Therefore, the observed FECs across the techniques resulted in concordance in classifying the intensity of infection from light to heavy according to the current WHO guidelines.

**Fig 5:**
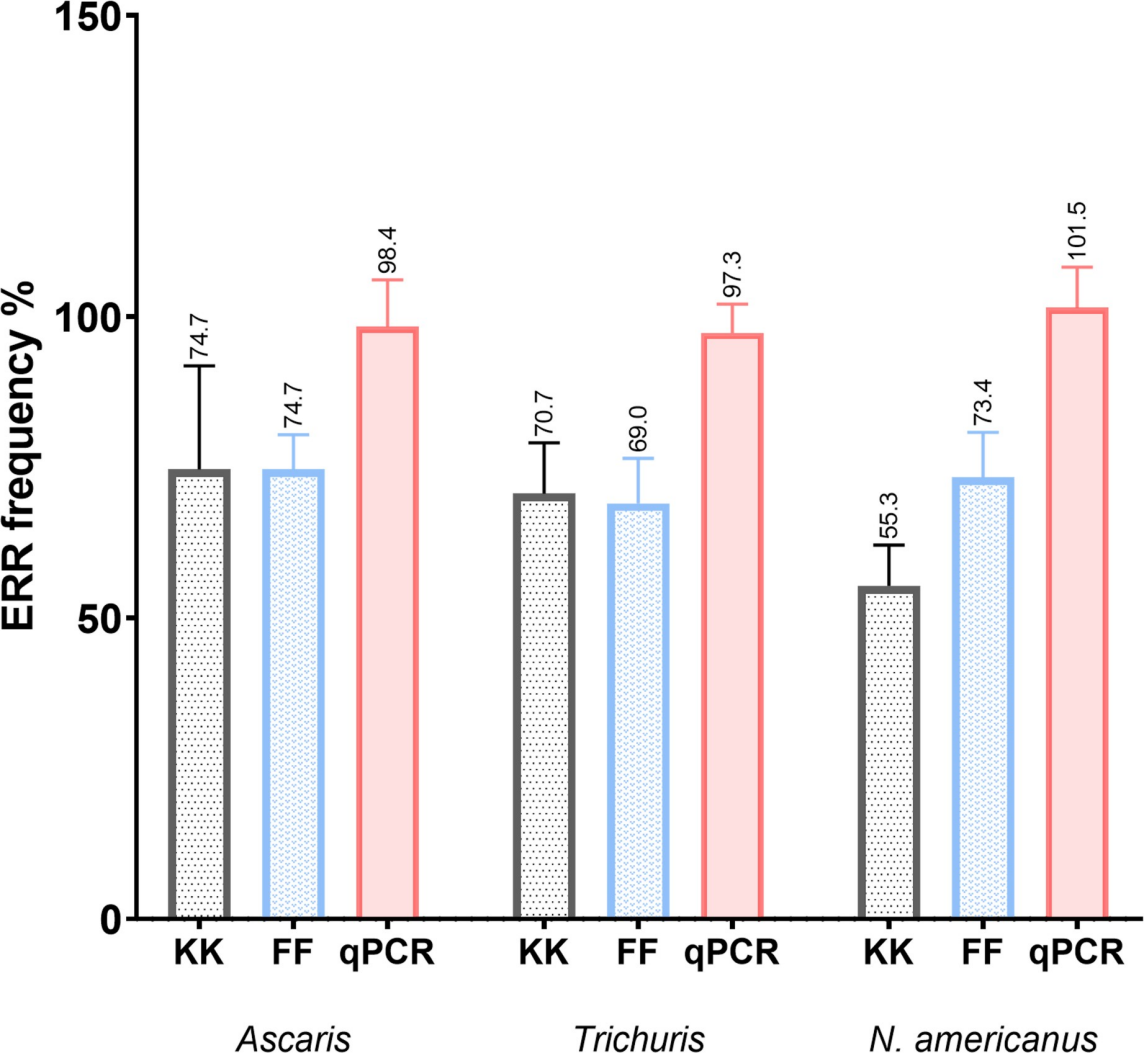
Egg recovery rate (ERR) frequencies of three diagnostic techniques. The bar plots represent the recovery percentage of each technique KK (Kato-Katz thick smear), FF (Sodium nitrate faecal floatation 1.30) and qPCR (quantitative qPCR) across the three most common STHs; *Ascaris*, *Trichuris* and *N*. *americanus*. Error bars represent standard error mean obtained from the overall ERR obtained from a series of known numbers of seeded egg experiments (Table 2).

Both KK and FF resulted in lower ERR as compared to qPCR (Fig 5). The overall mean ERR increased from 66.87% [95% CI: 41.48;92.27] with KK, to 72.36% [95% CI: 65.04;79.68] with FF, to 99% [95% CI: 93.61;104.5] with qPCR. ANOVA analyses revealed significant differences between the mean ERR of each technique for each parasite (F = 22.60 *p* 0.001)(Table 5). This result allowed to reject the null hypothesis that all means are equal. Because the overall test was significant, a post hoc test using Tukey’s multiple comparison test was conducted to compare the mean variation of each technique. The results indicated that KK and FF had no significant differences in ERR among parasites (*p* 0.564). However, KK vs qPCR and FF vs qPCR showed significant mean differences with *p* values of 0.001 and 0.004 respectively, contributing to the overall ANOVA significance difference (Table 5). Hence, qPCR outperformed microscopy by recovering around 1/3 (27% and 32%) more eggs than FF and KK respectively. FF and KK recovered eggs at a similar average frequency with FF recovering only 5.5% more eggs than KK.

**Table 5:** ANOVA- comparison of the egg recovery means among three diagnostic techniques.

**Source of variation**	**Sum of squares**		**Degrees of freedom**		**Mean sum of squares**	**F (DFn, DFd)**		***P value***
Variation between techniques	1777		2		888.6	F (2,6) = 22.60		0.001*
Variation within techniques	235.9		6		39.32			
Total	2013		8					
**Tukey’s multiple comparison test between techniques**		**Mean difference**		**95% Confidence interval**			**Adjusted *P value***	
KK vs FF		-5.483		-21.19 to 10.23			0.564	
KK vs qPCR		-32.17		-47.88 to -16.46			0.001*	
FF vs qPCR		-26.69		-42.40 to -10.98			0.004*	

α = 0.05*

### Limit of detection of microscopy and qPCR of low intensity infections

When low infection intensities were measured by means of qPCR, the lowest detection limit was 5 EPG corresponding to mean Ct values of 34.75 (S.D ± 0.22) for *Ascaris* spp., 35.19 (S.D ± 0.47) for *Trichuris* spp. and 35.42 (S.D ± 0.26) for *N*. *americanus* (Fig 2). In comparison, the limit of detection of FF from absolute egg counts was as low as 50 seeded eggs/gr for *Trichuris* (30 EPG), *Ascaris* (24 EPG) and *N*. *americanus* (39 EPG; Table 6). The limit of detection of the KK technique was the same as for FF (50 seeded eggs/gr), but EPGs resulted in comparable FECs for *Ascaris* (24 KK vs 42 FF EPG), *Trichuris* (12 KK vs 36 FF EPG), and *N*. *americanus* (12 KK and 39 FF EPG). According to WHO guidelines, all three techniques for all STHs can detect infections classified as light intensity infections. Nonetheless, qPCR outperformed by establishing light-moderate intensity cut-offs more accurately than FF and KK and by LOD for the three-main STHs.

**Table 6:** Seeded vs observed EPG counts for light-moderate cut-off infection intensities.

Seeded eggs per gram of faeces	Kato Katz observed EPG	NaNO_3_ Faecal float 1.3 SpGr observed EPG	M-qPCR observed EPG
***Ascaris* spp.**			
5	0	0	4
10	0	0	10
50	24	42	48
100	48	90	90
150	132	110	130
500	804	485	595
1000	1344	925	953
2000	3432	1513	2233
***Trichuris* spp.**			
5	0	0	5
10	0	0	13
50	12	36	44
100	96	74	66
200	168	163	179
500	360	395	438
***N*. *americanus***			
5	0	0	3
10	0	0	12
50	12	39	74
100	60	81	83
500	300	385	445
1000	660	825	865
1500	1008	1178	1311

## Discussion

To our knowledge this is the first study to accurately test the ERR and LOD of the KK, simple flotation and multiplex qPCR assays for STH eggs in human faeces.

Based on the ERR obtained from the graded SpGrs tested, a NaNO_3_ solution of SpGr 1.30 resulted in optimum recovery for *Ascaris*, *Trichuris* and *Necator* eggs in human stool. The SpGr of an egg is dependent on its volume and mass (water and solid content) [28]. Heavier eggs have a higher mass per unit volume and less per vitellus space in the egg. For parasite eggs to buoy up in a solution, the SpGr of the solute must be higher [29]. Common floatation solutions used in diagnostic practice include sodium/calcium chloride, sugar (Sheather’s solution), zinc sulphate (ZnSO_4_), magnesium sulphate and NaNO_3_. Nonetheless, compared to other solutions, NaNO_3_ has shown to be superior for the recovery of helminth eggs in canine faeces, including *A*. *caninum and Toxocara canis*. [17]. The data generated in this study supported those of previous work demonstrating that SpGrs of more than 1.20 deemed higher egg recovery rates in canine species [17,30,31]. Of importance, was the significantly superior ERR observed for *Trichuris* when using a SpGr of 1.30 (69.2%; 95% CI: 67.3;71.1) instead of the recommended SpGr of 1.20 routinely used for floatation techniques such as FLOTAC/mini-FLOTAC to detect parasites from human and animal faeces (6.5%, 95% CI: 5.26;7.74) [10,32].This observation may explain the lower prevalence observed of *Trichuris* spp. compared with qPCR [11,19] and FF [13] reported in previous epidemiological surveys and comparative studies in which a solution of SpGr of 1.20 was employed for flotation-based diagnosis of STH eggs. Failure to detect infections or underestimate the intensities of STH eggs has significant implications for accurately informing STH control programmes [33]. Previous studies have also supported our findings that allowing the cover slip to stand on the solution meniscus for less than 15 min prior to examination results in missed *Trichuris* infections in canine stool [17]. In addition to this, other variables such as the use of faecal preservatives, preservation times, egg developmental stage and ratio of different parasite egg species present in the faecal specimen, may influence egg recoveries even when using an optimal SpGr [29]. For example, Sawitz [28] tested the buoyancy of fertilized vs unfertilized *Ascaris* eggs showing that unfertilized eggs floated more readily in solutions of higher SpGr (1.25) when using ZnSO_4_ as compared to fertilized eggs which floated better in solutions of SpGrs of 1.20. Even though, ERR increased for *Ascaris* and *Trichuris* spp. using a SpGr of 1.35, this was not the case for *N*. *americanus*, that achieved optimum ERR at SpGr 1.30. Utilisation of a SpGr solution of 1.30 was deemed optimal as increasing this to 1.35 resulted in greater amounts of faecal debris and rapid crystallisation of NaNO_3_, which made eggs difficult to visualise. It must be noted that egg recovery of 100% is not achievable with any solution or SpGr as inevitably some eggs will adhere to the surface of the filter or may be attached within the faecal sediment [30]. To ensure floatation-based STH detection techniques perform optimally, investigators must ensure solutions attain correct SpGr and standing or centrifugation times are strictly adhered to when applying this technique.

A good correlation between multiplex qPCR assay Ct values and seeded EPG was obtained in agreement with previous experimental egg seeding studies demonstrating quantitation potential of the qPCR assays for enumeration of hookworm eggs in human faeces [14]. In addition, a strong direct correlation between the ERRs of qPCR, FF and KK for the experimentally seeded faecal samples were obtained across a range of egg intensities for all three STHs. Nevertheless, KK underestimated the seeded egg counts by 25–45% as compared to FF (~30%) and qPCR (2–3%). Both microscopy techniques performed equally for *Ascaris* and *Trichuris*, but not for *Necator* for which FF significantly outperformed KK in terms of ERR. This may be owing to the ability of hookworms to degrade and disappear in slides that are left to clear on KK smears for longer than 30–60 min, resulting in egg miscounts and/or false negative results, even though slide examination times were strictly adhered to in this study [13,23]. In accordance with previous studies [11] we observed a bell shape-like trend for the ERR for both coproscopy techniques, whereby light and very heavy intensity infections were underestimated, and moderate infections were more accurately quantified. In the case of light intensity infections, this may be due simply to the low sensitivity of detection microscopy techniques hold, which is well documented [10,12,34,35]. However, for heavy intensity infections, this may be explained by the superimposition of eggs by other eggs or faecal debris that may result in the underestimation of egg counts, in particular for the KK.

Our experimental data supports field epidemiological data demonstrating the superior sensitivity of molecular assays to that of coproscopic techniques for the detection of STH eggs in faeces [12,20,36–38]. For the detection and enumeration of STH eggs in faeces, the present qPCR assay performed best, exhibiting least ERR variation compared with double KK and FF. While a previous study [19] indicated that qPCR could not detect STHs in faecal samples with < 150 EPG, here we established that LOD was as low as 5 EPG for all three STHs studied. Other studies [11,19] have inferred EPG based on a calculation of genome equivalents per ml; such an inference is only valid if the genomic copy number(s) of each genetic marker used in qPCR is/are known, or can be estimated for developmental stages/sexes of STHs present in faecal samples [39]. For instance, it is well-known for nuclear ribosomal markers, such as internal transcribed spacers, that copy number can vary substantially among egg, embryonated egg, larvae and adult stages [40]; such variation can have a direct and significant impact on EPGs inferred from genome equivalents per ml, and on qPCR results. Similarly, copy numbers of ribosomal non-coding DNA markers may potentially vary between different strains of STH species.

In the present study, we employed EPG as an estimate of infection intensity, so that the qPCR results could be compared directly with conventional, quantitative coprodiagnostic methods, which follow current guidelines for the evaluation of faecal egg reduction after chemotherapeutic treatment. Nonetheless, there are still challenges that require further investigation to effectively incorporate qPCR as a diagnostic tool for STH diagnosis from field samples. For instance, repeatable standard operating procedures for DNA extraction and qPCR, effect of different stool preservatives and thermocyclers are still barriers that require attention [39].

Primary advantages of qPCR over coproscopic techniques include i) the ability to preserve faecal samples immediately in 100% ethanol for transport at ambient temperature to centralised laboratories for analysis; thereby negating the requirement for mobilising large teams of technicians and equipment to field sites that also require stable power supply and running water; ii) higher sensitivity and accuracy for detection and enumeration of STH egg intensities iii) ability to differentiate between species of hookworms that inform both morbidity and whether One Heath control measures are required.

To achieve and maintain elimination of STH morbidity in pre- and school aged children in context for WHO 2030 targets, prevalence with STH infections of moderate and heavy intensity must be lowered to <2%. Our study shows that microscopy techniques cannot accurately detect the cut-offs between light and moderate infection intensities by underestimating ERRs by approximately 20%, in particular when utilising KK for hookworm enumeration. This poses a challenge when programs aim to establish breakpoint of transmission, and more importantly, monitor recrudescence. If moderate infections are misclassified as light-infections by WHO, then MDA programs would be terminated too early.

## Conclusions

Compared to FF and to the WHO-recommended KK technique, only qPCR was able to accurately classify light-moderate infection cut-offs and to detect very low infection intensities, highlighting the suitability of qPCR to detect STH DNA in stool even in very low-infection settings. Further testing using clinical samples will be necessary to cross validate our results. Even though, KK is the only method that meets all the criteria for the planning, implementation and monitoring phase of MDA, the sensitivity of qPCR must be considered as a diagnostic tool by current programs aiming at maintaining infection levels at <2% as a threshold for MDA cessation and disease re-emergence monitoring.

## Supporting information

S1 File Standard operating procedure of sodium nitrate faecal float double cover slip. (PDF)
