# Supplementary material for: Comparison of the egg recovery rates and limit of detection for soil-transmitted helminths using the Kato-Katz thick smear, faecal flotation and quantitative real-time PCR in human stool
Source: PLoS Negl Trop Dis. 2021 May 26;15(5):e0009395. doi: 10.1371/journal.pntd.0009395 (PMC8153506; doi:10.1371/journal.pntd.0009395)
Supplement: S1 File — (PDF) [file pntd.0009395.s001.pdf]

Standard Operating Procedure

Sodium Nitrate Faecal Floatation double cover slip

Equipment to make up flotation solution:

Gloves

Stirring block

Stirrer/magnet if using a magnetic heating block

1-2 L beakers

Distilled water

Hydrometer

1 L Schott bottle

Sodium nitrate ($NaNO_3$)

Sodium Nitrate (SG 1.30)

~450 g $NaNO_3$ in 1 L dH_2O

Adjust SG to 1.30 SG by measuring with the hydrometer

Equipment and consumables for faecal flotation:

Non-sterile gloves – change between samples

Wooden ice-cream sticks

Bench top centrifuge that hold 15 ml centrifuge tubes (test tubes)

Permanent marker for labelling

Sturdy 10-15 ml tube rack

Sodium nitrate solution (SG 1.30) filled in a **250 ml** wash bottle

Surgical gauze (2 thin layers/sheets)

Non-sterile disposable 3 ml Pasteur transfer pipette

70 ml disposable specimen jar (non-sterile)

15/10 ml conical centrifuge tube (screw top)

Glass microscope slide

Cover slips 2 per sample (hydrophilic – those that attract water and not oil coated)

70% ethanol for clean up

1 L glass Schott bottles for chemical waste

Clinical/biological waste bin

Sharps bin

Method:

1. Place 2 layers of gauze onto the mouth of a 70 ml specimen jar
2. Mix faecal pellet well with pipette/ice-cream stick and place 1 gr/ml of faecal mixture onto the gauze
3. Holding gauze in place, strain faeces through gauze into specimen jar using 12 ml sodium nitrate solution
4. Squeeze remaining sodium nitrate solution out of gauze to jar using ice cream sticks
5. Mix and pour solution from jar into 15 ml centrifuge tube, rinse with 1 ml sodium nitrate to ensure no faecal sediment remains

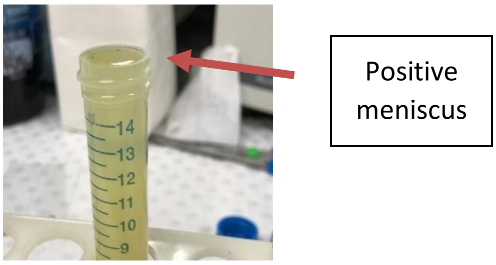

6. Top up to 14 ml mark with sodium nitrate solution
7. Spin for 5 min at 1800 x rpm
8. **Note the volume of the sediment (e.g. 400 µl or 0.400 ml)**
9. Remove and place upright on test tube rack

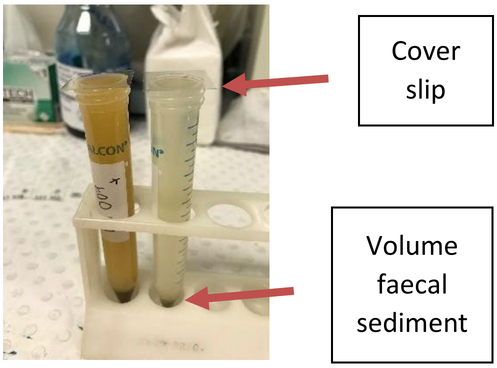

10. Continue adding $NaNO_3$ until positive meniscus forms
11. Wait for 10 min before placing the first cover slip
12. Carefully place coverslip on tube and leave for 5 min
13. Carefully remove coverslip ensuring drop of solution remains on the underside of the coverslip
14. Place coverslip on one side on microscope slide
15. Repeat step 12 using a second cover slip (5 min)
16. Place slide under the microscope and count all *Ascaris*, *Trichuris* and Hookworm eggs on coverslip 1.
17. If egg count < 1000, also count coverslip 2

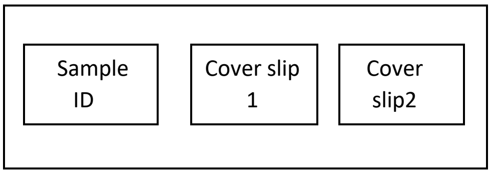

<u>To calculate eggs per gram of faeces use the following formula:</u>

If egg count coverslip 1 > 1000,

[1000/Volume of sediment (in µl)] * absolute egg count for each helminth (coverslip 1) *2

If egg count coverslip 1 < 1,000,

[1,000/Volume of sediment (in µl)] * absolute egg count for each helminth (coverslip 1 + coverslip 2)

Example 1: Volume of sediment 400 **µl** at step 8. Hookworm egg count coverslip 1 = 25, coverslip 2 = 35.

= 1,000/400 * 60 = 150 hookworm eggs per gram.

Example 2. Volume of sediment 600 **µl** at step 8. Ascaris egg count coverslip 1 = 1300;

= 1,000/600 * (1300 ***2)** = 4,333 Ascaris eggs per gram

Safety Precautions

Wear lab coat and disposable gloves

Wash hands thoroughly when finished

Cleaning Up Procedures

Pour sodium nitrate into appropriate chemical waste containers

Dispose of all disposable consumables and faeces in clinical or biological waste bin

Dispose all glass slides and coverslips into sharps container

Rinse any glassware and spray with 10% bleach or 70% ethanol prior to washing with water

Wipe microscopes down thoroughly as well as work area with 70% Ethanol

Sample reporting example:

Sample code	Faecal pellet volume (ml)	Absolute Egg Counts						Eggs per gram count		
		Ascaris		Trichuris		Hookworm		Ascaris	Trichuris	Hookworm
A001	300	2900	-	0	0	25	32	19,333	0	197
